# Supplementary material for: Shared Genetic Architecture between Muscle and Bone: Identification and Functional Implications of EPDR1, PKDCC, and SPTBN1
Source: bioRxiv. 2023 May 15:2023.05.14.540743. Preprint. [Version 1] doi: 10.1101/2023.05.14.540743 (PMC10245569; doi:10.1101/2023.05.14.540743)

Supplemental Material

S1 Fig. The summary per genomic risk locus. Note that genomic loci could contain more than one independent lead Single-Nucleotide Polymorphisms (SNPs).

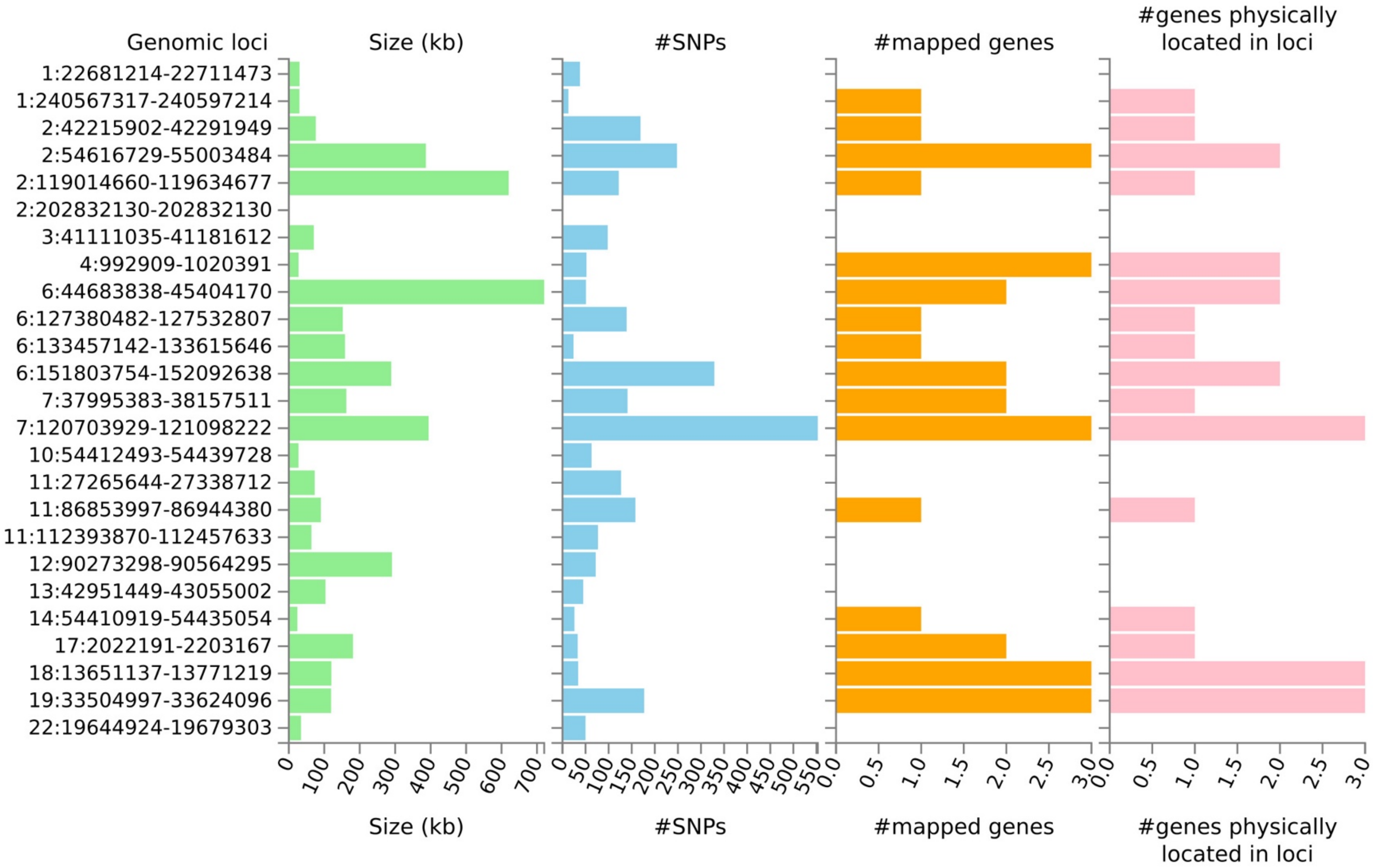

S2 Fig. **Tissue enrichment analysis using MAGMA** [20]. The most enriched tissue is the fallopian tube. Significantly enriched DEG sets ($P_{bon} < 0.05$) are highlighted in red. A threshold $P \leq 5 \times 10^{-8}$ was used to map the genes.

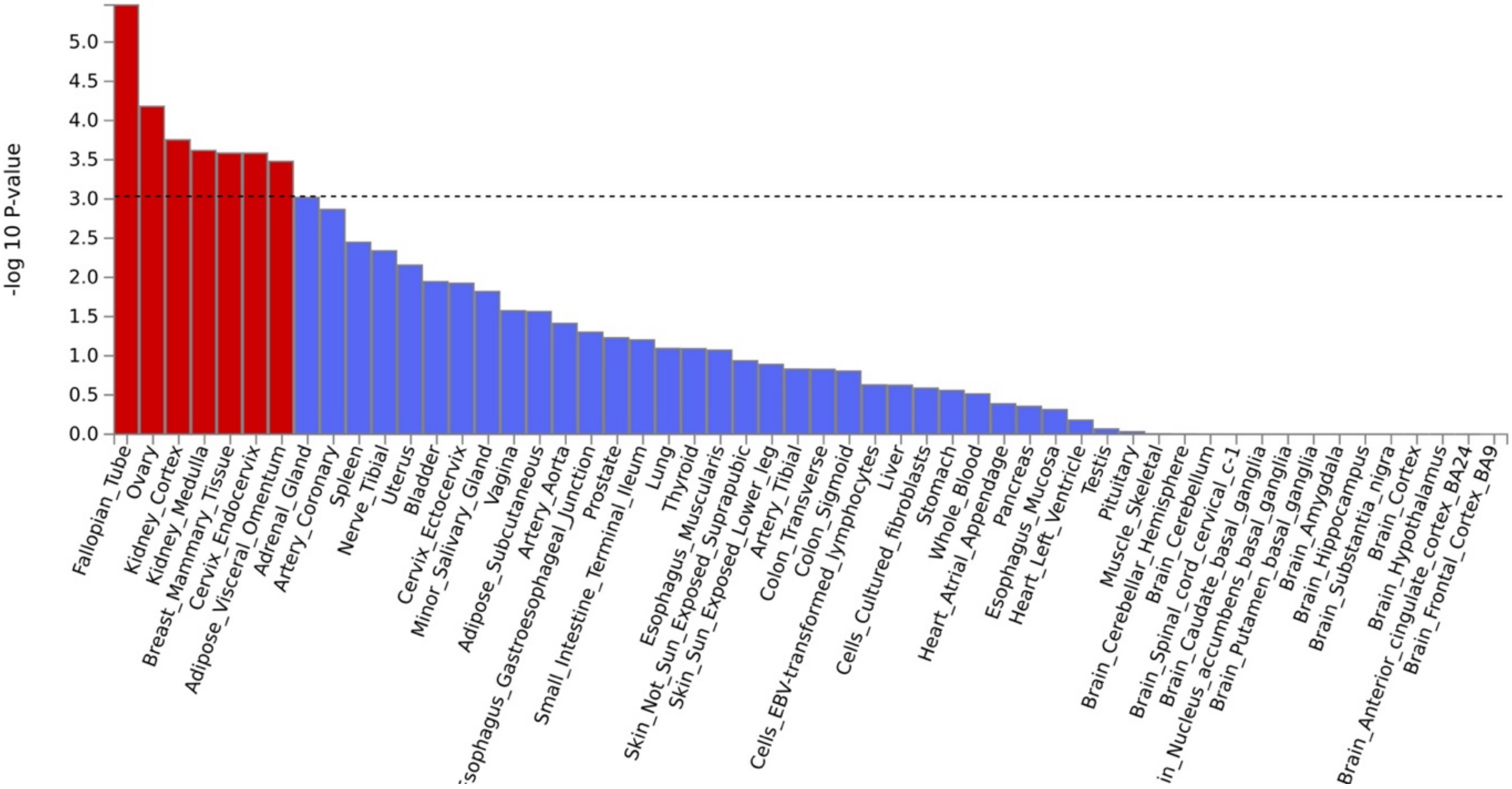

S3 Fig. **Differentially expressed genes in 30 major tissues in the GTEx data**. A threshold $P \leq 5 \times 10^{-8}$ was used to map the genes.

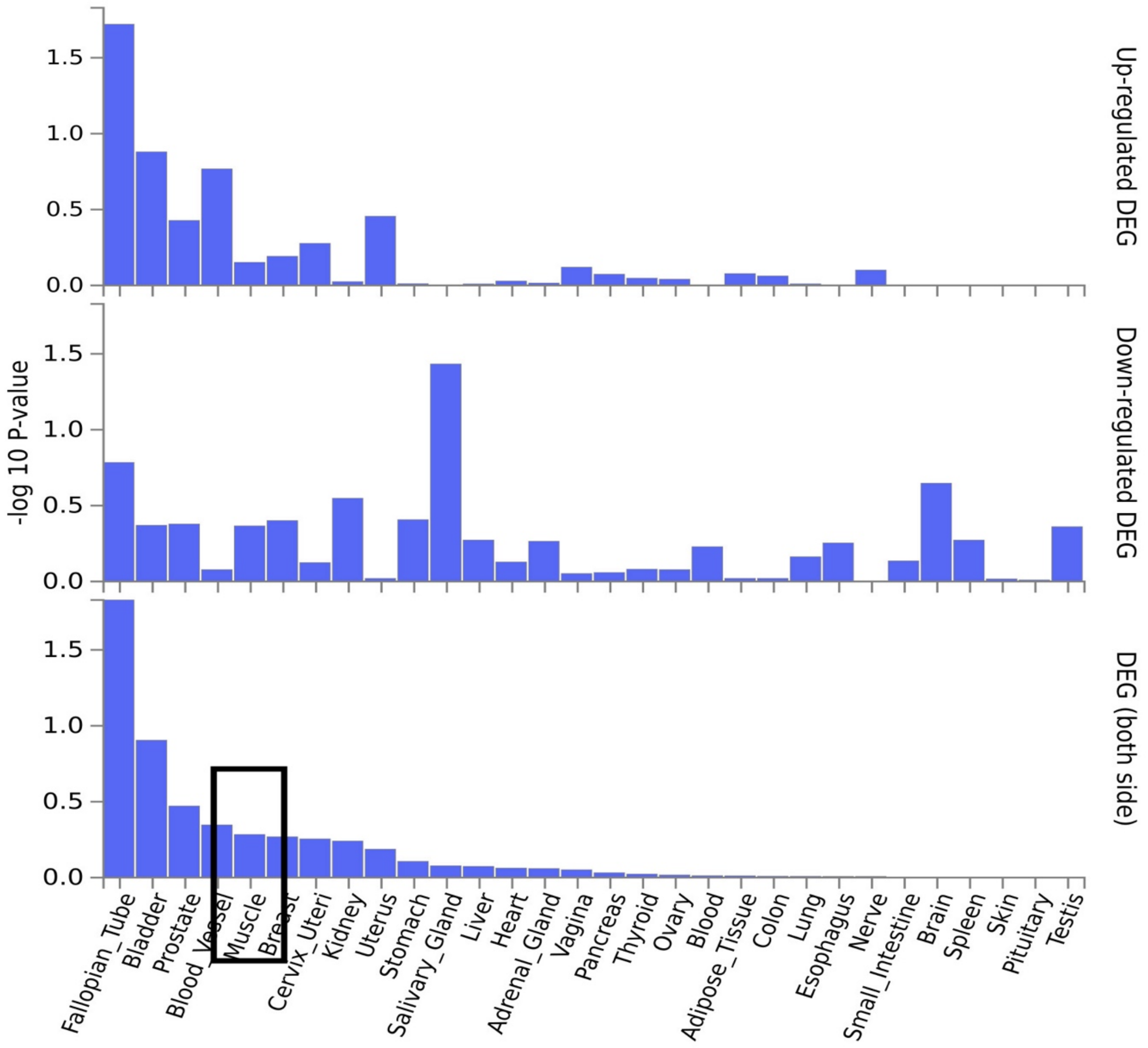

Supplement: Supplement 1 [file NIHPP2023.05.14.540743v1-supplement-1.pdf]
